# ESBLs: A Clear and Present Danger?

**DOI:** 10.1155/2012/625170

**Published:** 2011-06-06

**Authors:** Rishi H.-P. Dhillon, John Clark

**Affiliations:** ^1^Department of Medical Microbiology, Charing Cross Hospital, Imperial College Healthcare NHS Trust, Fulham Palace Road, London W6 8RF, UK; ^2^Epsom and St Helier University Hospitals NHS Trust, Wrythe Lane, Carshalton, Surrey SM5 1AA, UK

## Abstract

Extended spectrum *β*-lactamases (ESBLs) are enzymes produced by a variety of Gram negative bacteria which confer an increased resistance to commonly used antibiotics. They are a worrying global public health issue as infections caused by such enzyme-producing organisms are associated with a higher morbidity and mortality and greater fiscal burden. Coupled with increasing prevalence rates worldwide and an ever diminishing supply in the antibiotic armamentarium, these enzymes represent a clear and present danger to public health. This article aims to give an overview of the current situation regarding ESBLs, with a focus on the epidemiology and management of such infections.

## 1. Introduction and Definition

Critically ill patients are especially prone to infection, and the nature and epidemiology of causative agents can vary tremendously. In particular, drug-resistant pathogens are of a major concern, as they carry a higher morbidity and mortality and are more difficult to identify by routine laboratory assays, which can lead to a delay in diagnosis and institution of appropriate antimicrobial therapy. There is also a growing concern regarding the lack of new antibiotics [1] especially for multidrug-resistant Gram-negative bacteria which produce extended spectrum *β*-lactamases (ESBLs). 

In June 2010, the Infectious Diseases Society of America gave testimony before the House Committee on Energy and Commerce Subcommittee on Health, on the critical need for stewardship of antimicrobials and the urgent necessity of research and development into newer therapies.

*β*-lactamases are hydrolytic enzymes which cleave the *β*-lactam ring and are the primary mechanism of conferring bacterial resistance to *β*-lactam antibiotics, such as penicillins and cephalosporins.

These enzymes can be carried on bacterial chromosomes, that is, inherent to the organism, or may be plasmid-mediated with the potential to move between bacterial populations. This has clear implications regarding spread of infection and infection control, which will be discussed later.

ESBLs are primarily produced by the Enterobacteriaceae family of Gram-negative organisms, in particular *Klebsiella pneumonia *and *Escherichia coli* [2, 3]. They are also produced by nonfermentative Gram-negative organisms, such as *Acinetobacter baumannii* and *Pseudomonas aeruginosa *[4]. 

The original method of *β*-lactamase categorisation is the Ambler classification [5] which orders the enzymes into 4 classes (A, B, C, and D) based on molecular structure. 

ESBLs are Class A *β*-lactamases and may be defined as plasmid-mediated enzymes that hydrolyse oxyimino-cephalosporins, and monobactams but not cephamycins or carbapenems [6]. They are inhibited in vitro by clavulanate [2]. 

There are various genotypes of ESBLs. Of these, the most common are the SHV, TEM, and CTX-M types [7]. Other clinically important types include VEB, PER, BEL-1, BES-1, SFO-1, TLA, and IBC [4]. 

In 1995, Bush et al. devised a classification of *β*-lactamases based upon their functional characteristics and substrate profile, a classification which is widely used [8]. 

The enzymes are divided into three major groups: group 1 cephalosporinases which are not inhibited by clavulanic acid, the larger group 2 broad spectrum enzymes which are generally inhibited by clavulanic acid (except for the 2d and 2f groups) and the group 3 metallo-*β*-lactamases.

The main points are illustrated in Table 1. 

Most ESBLs are assigned to group 2be, that is, hydrolyse penicillins, cephalosproins, and monobactams, and inhibited by clavulanic acid (as per the Ambler classification). It should be noted that the CTX-M genotype was not classified in this original schemata but still fulfils the above criteria for group 2be enzymes.

## 2. Epidemiology

When ESBLs were first recognized in the early 1980s, they were found to be point mutations of the TEM and SHV broad spectrum enzymes, which resulted in resistance to the *β*-lactam class of antibiotic [9, 10]. The mutations in the genes results in these enzymes having high catalytic capabilities for *β*-lactams due to low *K*_*m*_ values (i.e., high affinity) for the compounds [11]. 

They have become a major cause of hospital-acquired infection, particularly in the intensive care unit (ICU), with the majority of ESBL producers being isolated from critical care patients [3, 12]. 

TEM and SHV-types have been recognized across the world with over 100 mutations being reported as offering resistance to the extended spectrum cephalosporins. This was driven by the heavy use of such antibiotics [13]. 

Since the start of the 21st century, it has becoming increasingly evident that a shift in the genotypic makeup of ESBLs is taking place. 

The CTX-M genotype, originating from chromosomally encoded enzymes of the *Kluyvera spp*, has risen in prominence especially in *E. coli *and* K. pneumonia *[3, 12, 13]. It is believed the genes were then conjugated onto plasmids from where they were transferred to pathogenic species, with the ability to move between different bacterial populations [14].

 The CTX-M enzymes appear to have a greater ability to spread and cause outbreaks.

There are over 50 variants of CTX-M to date, and they have been associated with numerous outbreaks of infections both in hospitals and in the community, particularly in urinary *E. coli* isolates in a nonhospital setting [3, 13]. 

A paper two years ago in the Lancet Infectious Diseases described some key characteristics between hospital and nosocomial infections with ESBLs [15].

This includes the informations mentioned in Table 2.

Risk factors for acquiring hospital associated ESBL infection [16] include the informations mentioned in Table 3.

Risk factors for acquiring community-associated ESBL infection [17] include:

- recurrent UTI,
- previous antibiotic usage,
- diabetes mellitus,
- prior instrumentation to urinary tract,
- female sex,
- age (over 65 years).

Data from the last 10 years establishes CTX-M genotype as the predominant ESBL in Europe and East Asia, as will be discussed below.

Clearly, the rise of multidrug-resistant organisms in the community is of a huge public health concern.

### 2.1. Global Epidemiology

The prevalence of bacteria producing ESBLs varies worldwide, with reports from North America, South America, Europe, Africa, and Asia [14]. Data from the Tigecycline Evaluation and Surveillance Trial (TEST) global surveillance database shows the rate of ESBL production was highest among the *K. pneumoniae* isolates collected in Latin America, followed by Asia/Pacific Rim, Europe, and North America (44.0%, 22.4%, 13.3%, and 7.5%, resp.)[3, 18].

### 2.2. USA

First reports of ESBLs in the USA in the late 1980s were reported with TEM-type [19] and the major enzymes appear to be the TEM and SHV types, with a minimal appearance of CTX-M types [20]. Both the prevalence of ESBLs and types involved found in the USA are in stark contrast to the epidemiology seen in the rest of the world, including Canada, where outbreaks of CTX-M producing *K. pneumoniae* have been seen [21]. 

The juxtaposition of the USA and Canada would suggest the spread of CTX-M types south of the border is a real threat. Indeed a recent report draws attention to the emergence of CTX-M types in a US health care system [22]. 

Data from several surveillance surveys conducted in the USA provides a broader picture of prevalence rates [20]. 

The National Nosocomial Infections Surveillance Systems report issued in October 2004 from the CDC compared data on nosocomial infections from several centres across the United States [23]. It looked at ICU and non-ICU settings.

Rates of *K pneumonia* resistant to third generation cephalosporins (i.e., presumed ESBL producers) increased by 43% in 2003, compared with data from 1998 to 2002. Resistant *E. coli* rates were unchanged. When resistant rates were pooled across all ITUs (to include adult, paediatric, and cardiothoracic), the percentage of resistant *K pneumonia* accounted for 6.2% of isolates, whereas cephalosporin resistant *E. coli* was lower at 1.3% of all *E coli* isolates from ITU patients. This compared to 5.8% and 1.5%, respectively, in non-ITU settings. 

In the late 1990s, the SENTRY study analysed isolates over a 12-month period in the late 1990s and showed a higher percentage of ESBLs in *K. pneumoniae* strains in US centres versus Canadian (7.6% versus 4.9%), with *E. coli* producing ESBLs demonstrating no significant difference [24].

Interestingly More Recent Data from the MYSTIC programme (which looked at ESBL production from *K. pneumoniae* and *E. coli* isolates over a 5-year period from 1999–2004) showed a low level of ESBL prevalence, with less than 1.5% of *E. coli* strains producing ESBLS from 2001–2004. A similarly low level was seen in *Klebsiella spp* over the same time period, 2.4%–4.4% of strains producing the enzymes [25].

These low numbers are further corroborated by data from the CDC which looked mainly at hospital acquired infections, with ESBLs contributing to 0.5%–1% of hospital acquired infections [26].

### 2.3. Europe

In Europe, ESBL-producing Enterobacteriaceae has been spreading at an alarming rate. Although there is extensive difference between European countries, almost every European country has experienced outbreaks with ESBL-producing organisms [2].

The first isolates were originally detected in Germany [9] and the UK; however, the first large outbreak was seen in France, where over 50 patients in an intensive care unit were affected with spread to other wards in the hospital [27]. 

During the 1980s and 1990s, TEM and SHV were the predominant ESBL type. They were almost exclusively hospital acquired infections and were associated with nosocomial outbreaks, particularly in the ICU [28]. This role of the ICU as a source of ESBL outbreak is commonly recognized as part of the epidemiology of these organisms [3, 12]. 

**Figure 1:**
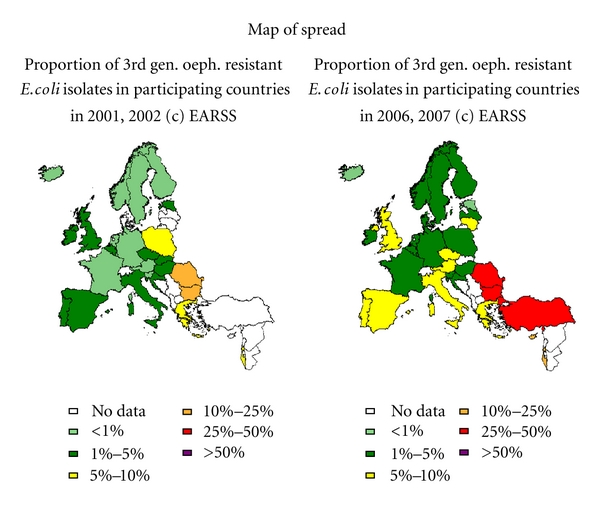


Data from the European Antibiotic Resistance Surveillance System confirms the increasing prevalence of ESBLS across Europe (see Figure 1).

Interestingly, there has been a slight fall in the number of *K. pneumoniae* producing ESBL in Western Europe, likely due to enhanced infection control practices and antimicrobial stewardship [29]. However, this is not true for Eastern Europe, where numbers of resistant isolates appear to rising [30]. According to the annual epidemiological report on communicable diseases in Europe 2010 [31], *E. coli* showed a Europe wide increase in resistance to all antibiotics under surveillance.

The striking proliferation of the CTX-M enzymes has resulted in a change in the distribution of ESBL types across Europe; currently, CTX-M and TEM are the main types [32]. In addition, community-acquired ESBL producing organisms causing urinary tract infections, especially *E. coli*, are showing a worrying rise in numbers [28].

It has been consistently shown that rate of ESBLs in Europe is higher of that in the USA but lower than in Latin America and Asia.

### 2.4. South America

Rates of ESBLs in South America rank amongst the highest in the world, with CTX-M dominant. Surveillance data reveal alarmingly high prevalence rates, with *Klebsiella* isolates producing ESBLs from Latin America ranging from 45% to 51% [24, 33]. 

Similarly, high rates are seen amongst *E. coli* isolates in Latin America ranging from 8.5% to 18% [33].

There are many reasons as to why prevalence rates should be so high in this part of the world. Certainly, there is ample evidence to suggest the spread of ESBL infections is higher in resource poor countries [34, 35]. 

This could be due to poorer social and economic situations, hospital overcrowding, lack of antimicrobial stewardship and excessive over the counter antibiotic usage and undersupported infection control practices [36].

### 2.5. Asia

In Asia, high rates of ESBL producing Enterobacteriaceae are seen.

This was first highlighted by the SENTRY antimicrobial surveillance programme 1998-1999 [37]; data prior to this is lacking. Clearly over such a large geographical area, a large variation is seen in prevalence rates and genotype of ESBL. For example, in China, the incidence of ESBL production from *E. coli* isolates ranged from 13%–15%, with even higher rates amongst *Klebsiella* (>20%, with one centre reporting over 60%) [37]. 

The paucity of data prior to the late 1990s makes it difficult to ascertain the genotypic makeup of the ESBL producers during that time. The first reports from Japan and Taiwan suggest the SHV type played a key role early on but, just like in mainland Europe, the rise of the CTX-M genotype has made it the preeminent enzyme, with national and regional variations [38].

### 2.6. Africa

In comparison with the rest of the world, there is generally a lack of comprehensive data regarding ESBL producing Enterobacteriaceae in African countries. However, there is sufficient evidence to highlight the prevalence of ESBLs in Africa.

It is recognized that Egypt has an extremely high rate of ESBL producers, with up to 70% of isolates producing the enzyme [39]. 

One survey compared data from Egypt, Lebanon, Saudi Arabia, and South Africa, and Egypt was found to have the highest rates of ESBLs [40]. 

Again, the CTX-M genotype appears to be the most common type in North Africa [41]. There have also been reports of CTX-M *K. pneumoniae* in Kenya [42] and SHV and TEM—types in South Africa [43]. 

The high rate of ESBL producers in the developing world is clearly worrying; lack of funds for effective infection control and limited access to effective antimicrobials has clear implications with regards to curbing the morbidity and mortality associated with these infections.

## 3. Clinical Implications

There is no doubt that ESBL-producing organisms are of enormous clinical and microbiological significance.

Such bacteria are associated with severe infections such as bacteraemias, intra-abdominal infection, urinary tract infections (particularly in the community setting), and respiratory tract infections [15]. 

They inactivate cephalosporins, which are often used in treating the septic patient in a variety of clinical settings. Therefore, this often renders empiric antibiotic treatment ineffective. The delay in laboratory diagnosis and time to appropriate antibiotic therapy has been strongly linked to an increased mortality in these cases [44, 45].

Therefore, it is of paramount importance that local surveillance data of prominent infective pathogens is closely monitored.

As previously mentioned, many ESBL genes have the propensity to jump between organisms, thus leading to outbreaks of infection if this occurs in an easily transmissible pathogen.

It is also known that organisms producing ESBLs also have the ready capacity to acquire resistance to other antimicrobial classes such as the quinolones, tetracyclines, cotrimoxazole, trimethoprim, and aminoglycosides, which further limits therapeutic options [12, 46–48]. 

The mechanism behind this multiresistance phenomenon is genetic; the gene encoding for resistance for both ESBL and other classes (e.g. quinolones) are often associated on the same mobile DNA element (plasmid) [28]. The propagation of this plasmid during conjugation leads to development of multidrug resistance in previously sensitive organisms.

## 4. Laboratory Diagnosis

The laboratory diagnosis of ESBL-producing bacteria is complex and the intricacies are beyond the scope of this paper.

Essentially, most clinical diagnostic laboratories detect ESBL producers by phenotypic tests, which require a screening step followed by confirmation.

The screening test is based on testing the organism for resistance to an indicator cephalosporin. Cefpodoxime is commonly used as it is hydrolysed by TEM, SHV, and CTX-M types, but other cephalosporins such as cefotaxime, ceftriaxone, and ceftazidime are also used [49]. 

To confirm the presence of an ESBL, synergy between the indicator cephalosporin and clavulanic acid needs to be demonstrated (ESBLs are inhibited by clavulanic acid) [49]. 

**Figure 2:**
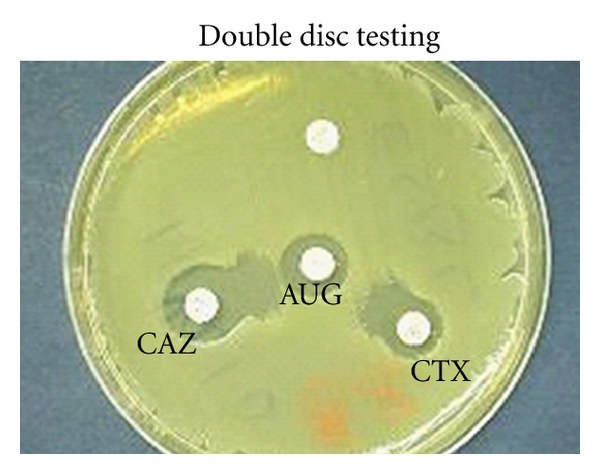
Synergy is seen as an expansion of the cephalosporin zone adjacent to the clavulanate containing disc CAZ—good substrate for TEM and SHV's, CTX—Good for CTX-M ESBLs. The organism may appear resistant to 3rd-generation cephs, but susceptibility is restored by the presence of clavulanate.

**Figure 3:**
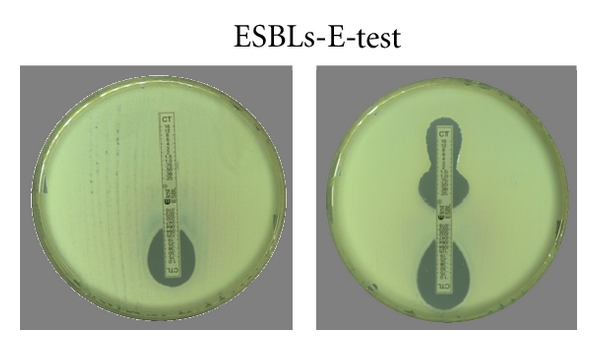
MICs that are >8 fold lower with clavulanate (left) or the presence of keyhole zones (right) imply ESBL production.

There are a variety of commercial tools available to do this, including double disc synergy, combination disc method, and specific ESBL *E*-tests [49, 50] (see Figures 2 and 3). 

However, if the isolate produces an additional AmpC or metallo-*β*-lactamase (which are not inhibited by clavulanic acid), these methods will lose their sensitivity [50]. 

Both screening and confirming the presence of an ESBL producer can be technically difficult, and it is time consuming. This can be a significant clinical problem, as time to appropriate antibiotic is crucial in the management of a septic patient.

Reference laboratories can test for genes encoding ESBLs by molecular analysis, primarily polymerase chain reaction amplification of specific sequences. This is usually reserved for epidemiological purposes, as it identifies the particular genotype of ESBL [15]. 

Newer technologies such as the molecular techniques above and modifications of mass spectrometry (matrix-assisted light desorption ionisation time-of-flight; MALDI-TOF) are being mooted as quicker alternatives to conventional laboratory diagnosis. However, these technologies are still relatively new in development and are not for use in most clinical institutions.

## 5. Management

With regards to the antimicrobial treatment of the septic patient, there is a lack of options against ESBL-producing organisms.

As well as hydrolysing the *β*-lactam ring found in penicillin, cephalosporins (except cephamycins), and aztreonam, ESBL producers often have other mechanisms that confer resistance to other classes of antimicrobials, as described earlier.

### 5.1. Carbapenems

Carbapenems are regarded as the antibiotic of choice and mainstay against severe infections caused by ESBLs [3, 15]. 

They are rapidly bactericidal and demonstrate time-dependant killing. They are stable against the hydrolytic activity of the enzyme and although most effect has been shown in in vitro studies, there is certainly enough data to support its clinical efficacy [51–53]. 

They also have the added benefit of being effective against other classes of *β*-lactamases such as the Amp C class. In the UK, Imipenem, Meropenem, and Ertapenem are the major drugs available in this class and generally have equal efficacy against most bacteria [54, 55]. However, there is some data to suggest Ertapenem is more susceptible to resistance than the other two [56]. Doripenem, is a newer carbapenem and is licensed for use in several countries (including Japan, USA, and in Europe) for treatment of severe bacterial sepsis. Like the other carbapenems, it is stable against ESBL producing organisms and is considered to have greater efficacy against *Pseudomonas aeruginosa *[57]. 

Resistance to carbapenems has been seen in some strains of *Klebsiella* and *E. coli* species, in the form of carbapenemases (*Klebsiella* producing carbapenemases (KPC) and New Delhi metallo-*β*-lactamases (NDM)) and there is an increasing concern on the overreliance on carbapenem therapy [52, 58–60].

### 5.2. Fluoroquinolones

If the ESBL producing organism is sensitive to ciprofloxacin in vitro, a good clinical outcome can be achieved using quinolones [58]. In UTIs caused by susceptible ESBLs, quinolones may be regarded as an excellent treatment option [2].

However, the empirical use of fluoroquinolones to treat these infections is generally not recommended, due to the concern of resistance, the rates of which are increasing worldwide [58]. This is particularly the case in serious infections. Two large studies compared the efficacy of carbapenems over quinolones in treating *K. pneumoniae* bacteraemia. One favoured carbapenems and the other found equivalent efficacy [61, 62]. 

### 5.3. Aminoglycosides

As per the quinolones, if an organism is susceptible on antibiotic testing to aminoglycosides, they are effective. Aminoglycosides can be a useful adjunct due their rapidly bactericidal activity; however, their use as monotherapy should be avoided where possible particularly in serious infection [63]. 

### 5.4. Fosfomycin

Fosfomycin has excellent in vitro activity against ESBL-producing Enterobacteriaceae [64]. 

It is one of the few antibiotics active against ESBLs that can be administered orally. It has been proven effective against susceptible ESBL producing isolates causing cystitis [17]. 

Work has also been done looking at its use for nonurinary and gastrointestinal tract infections [65]. This study showed high cure rates of ~80%, but it looked at the overall use of fosfomycin against all bacteria, and the drug was often used in conjunction with other antibiotics and other management modalities, for example, surgery. With particular regard to its activity against ESBL producers, fosfomycin is a viable option in urinary tract infections especially as resistance appears to be low at present [17, 65].

### 5.5. Tigecycline

Tigecycline is a derivative of minocycline with a broad spectrum of activity. It has excellent in vitro activity against ESBL producers, especially *E. coli* isolates, but data reflecting clinical outcomes is lacking [66].

A drug safety communication from the FDA in September 2010 (http://www. fda.gov/Drugs/DrugSafety/ucm224370.htm) warned against its use against serious infections, in particular against its use for HAP/VAP. This was due to an increase in mortality in Tigecycline treated patients compared with other antibiotics, possibly due to its bacteriostatic mode of action.

### 5.6. Cephalosporins

Generally, cephalosporins are not recommended treatment for ESBL infections as these enzymes inactivate the drug even if in vitro antibiotic testing reports a susceptible organism [67]. Indeed such isolates should be reported as resistant. Studies have looked at Cefepime as a potential therapeutic option, but clinical data does not support its use, with high failure rates and inferiority compared to carbapenem therapy [67–69]. 

Relevant clinical data regarding the use of cephamycins is scarce and there is considerable concern over its efficacy in this situation, mainly due to coresistance [70].

### 5.7. *β*-lactamase Inhibitor Combinations

These agents may be active against organisms possessing a single ESBL [2]. 

Whilst it should never be used for serious infections, amoxicillin/clavulanate may be effective in community acquired UTIs caused by susceptible ESBLs [17, 71].

Tazobactam has been shown to be more effective against CTX-M ESBLs compared with clavulanate whilst both appear superior to sulbactam against SHV and TEM types [72, 73]. 

However, this is rarely useful as genotypic testing is not performed in clinical laboratories. Again, clinical data on the use of these drugs against ESBLs is lacking and using these agents would not be appropriate in serious infections [3, 74].

### 5.8. Polymixins (Colisitin and Polymixin B)

Colistin is often used to combat multidrug-resistant organisms, in particular *Acinetobacter baumannii* and *Pseudomonas aeruginosa *[75]. 

It has excellent efficacy against ESBL producers [76] and with the emergence of carbapenem resistant organisms (e.g., KPCs), it has been used (albeit rarely) to treat such infections and curb outbreaks [77].

### 5.9. Nitrofurantoin

Nitrofurantoin can be effective in uncomplicated UTIs caused by ESBL producers [78].

### 5.10. Temocillin

Temocillin is a derivative of Ticarcillin and is licensed for use in the UK and Belgium for serious infections caused by susceptible organisms. It is stable to *β*-lactamase action and, therefore, active against all SHV, TEM, and CTX-M ESBLs and AmpC *β*-lactamases, making it an excellent alternative to carbapenems in sensitive bacteria [79]. It is well tolerated and appears to have little potential to select for *C. difficile *[80, 81]. 

The major drawbacks of Temocillin is its lack of activity against Gram-positive organisms, anaerobes, and *Pseudomonas aeruginosa *[82, 83]. 

However, in the battle against ESBL-producing infections there is compelling evidence to suggest that Temocillin is an extremely useful agent [79]. As with a lot of the antibiotics discussed here, clinical outcome data is scanty and dated. However, the experience from Belgium, where it has been in clinical use for over 6 years, provides evidence that it is effective in serious infections, especially hospital acquired pneumonia, caused by susceptible ESBL producers [79].

## 6. Infection Control

### 6.1. Hospital Cases

ESBL producing organisms can spread easily within the hospital environment. Most commonly, the transient carriage of organism on the hands of health care workers are implicated in patient to patient spread [84–86].

Environmental contamination is also a potential source with sinks, baths, and medical equipment such as bronchoscopes, blood pressure cuffs, and ultrasound gel all being reported as sources of infection [2]. 

Small hospital outbreaks tend to be caused by a single clone and usually occur in high risk areas such as the ICU, neonatal units and haematology-oncology units [87–89].

Large outbreaks usually involve several circulating strains of organism at one time and affect several areas in a healthcare setting.

Effective infection control requires a multidisciplinary approach and the principles are the same as with tackling any multidrug resistant organism.

Preventing spread of such organisms from patient to patient is the main focus of infection control measures. The main issues are hand hygiene of healthcare professionals, use and cleaning of medical equipment, and colonisation of the environment [2]. 

 Correct hand hygiene and an adequate level of nursing staff are crucial in order to reduce the risk of spread between patients. Screening is advocated in patients being admitted or transferred from other institutions, including nursing and residential homes [90]. Surveillance of infected and high risk patients is used to either monitor an outbreak or, preferably, prevent one. In this case, rectal swabs on selective media are used [2]. 

Patients who are infected with such infections should be nursed in a single room, or cohorting may be necessary if such isolation facilities are limited [90]. 

Antimicrobial stewardship is of paramount importance, especially in this era of increasingly resistant organisms, coupled with a lack of antimicrobial options. Selection pressure must be avoided by judicial and prudent use of antibiotics. In the context of ESBL producers, a variety of antibiotic classes must be considered a risk for inducing selective pressure, not just *β*-lactams and cephalosporins [88, 91] although limiting widespread use of third generation cephalosporins has been shown to be effective in limiting ESBL producers [92]. Some data supports the switch of cephalosporins to piperacillin/tazobactam to try and curb the rising rates of such organisms [92, 93].

The danger with this strategy is the emergence of further drug-resistant organisms.

The use of fluoroquinolones can also select for ESBL producers, as the gene encoding for resistance for both ESBL and quinolones are often found on the same mobile DNA element [28]. This again reinforces the importance of recognising local resistance patterns. An antibiotic policy which takes this into account and restricts the use of broad spectrum agents (especially third-generation cephalosporins) is well recognised as key [28, 90, 92, 93]. 

Clearly, treating the infected patient is crucial in limiting the spread of ESBLs and antibiotic options have been discussed above. It should be mentioned that the CTX-M type are increasingly associated with other resistance mechanisms which further limits options. This has an obvious impact on community outbreaks [12]. 

Certain medical procedures increase the risk of infection by promoting translocation of these organisms form colonising areas. Gastrointestinal surgery, intubation, and urinary catheterisation are all associated with this occurrence [90, 94]. 

The use of selective decontamination in the infected patient remains controversial. Although it may be effective in reducing the risk of spread, resistance (as well as other adverse effects associated with antibiotics) to therapies used in this context is a major problem [2, 95]. 

In an outbreak situation, it is important to establish whether the infection is caused by the same clone (oligoclonal) or by multiple clones (polyclonal) of the organism. Oligoclonal outbreaks imply horizontal transfer, that is, person-person spread of the same bacteria, whereas polyclonal outbreaks may be caused by selective antibiotic pressure [2]. This useful information allows the institute to focus its control measures appropriately. Molecular analysis by reference laboratories allows the clones to be identified. 

Therefore, one can look at controlling hospital outbreaks at both the individual and institutional level. 

In dealing with the infected patient, priority must be given to appropriate and effective antimicrobials, good hand hygiene, and avoiding unnecessary procedures, including central venous catheters. 

On an institutional level, screening and isolating all such infected patients with appropriate infection control practices, restricting use of broad spectrum cephalosporins across the hospital (i.e., implementing a stricter antimicrobial policy) and investigating environmental contamination are important [96, 97]. 

### 6.2. Community Cases

Community outbreaks can produce different challenges. As mentioned, these tend to be CTX-M producing *E. coli*, frequently urinary tract infections. One paper [90] looked at outbreaks of CTX-M producing *E. coli* infections in the UK in 2002-2003 and found a variety of clones, plasmid transmissibility, and phenotypic behaviour, all of which can impact on any infection control measure. As well as the traditional strategies described above, the authors concluded thorough investigation and termination of food sources (raw meats are commonly implicated), scrutinising any ongoing environmental risks and screening of high risk admissions to health care facilities are all important in preventing spread of community acquired infections.

## 7. The Future

There is no doubt that ESBL-producing infections are of grave concern to the medical world. They are associated with an increased morbidity and mortality and can be difficult and time consuming to identify. Coupled with the fact that prevalence rates are rising globally, including in nonhospital settings, and the dire lack of effective antimicrobial therapy, the future is tremendously concerning. Urgent work is required to develop quicker, cost-effective, and reliable diagnostic tools as well as new effective therapies.

## Figures and Tables

**Table 1:** 

Bush-Jacoby-Medeiros Group	Molecular class (Ambler)	Preferred substrates	Representative enzymes	Resistance or susceptibility to *β*-lactamase inhibitors
1	C	Cephalosporins	AmpC	Resistant
2b	A	Penicillins, Cephalosporins	TEM, SHV	Susceptible
2be	A	Penicillins, extended-spectrum cephalosporins, monobactams	TEM, SHV	Susceptible
2d	D	Penicillins, cloxacillin	OXA	Resistant
2e	A	Cephalosporins	Inducible cephalosporinases from *Proteus vulgaris *	Susceptible
2f	A	Penicillins, cephalosporins, carbapenems	NMC-A from *Enterobacter cloacae *	Resistant
3	B	Most *β*-lactams including carbapenems	L1 from *Stenotrophomonas maltophilia *	Resistant

Amended from original Bush-Jacoby-Medeiros classification scheme for bacterial *β*-lactamases.

**Table 2:** 

	Community onset	Hospital onset, particularly ITU
Organism	*E. coli*	*Klebsiella spp*
Type of ESBL	CTX-M	SHV,TEM
Type of infection	Usually UTIs, but also bacteraemia and GI infection	Bacteraemia, intra-abdominal, and respiratory and urinary infection
Molecular epidemiology	Isolates not always related	Isolates usually related, that is, outbreak

**Table 3:** 

Factor	Odds ratio (95% CIs)
ICU admission	1.67 (1.16–2.40)
Renal failure	1.92 (1.21–3.04)
Burns	2.78 (1.92–4.01)
TPN	1.72 (1.18–2.49)
Urinary catheter	1.88 (1.25–2.83)
3rd Gen cephalosporin	2.99 (1.6–4.0)
